# Reciprocal Regulation of Substance P and IL-12/IL-23 and the Associated Cytokines, IFNγ/IL-17: A Perspective on the Relevance of This Interaction to Multiple Sclerosis

**DOI:** 10.1007/s11481-015-9589-x

**Published:** 2015-02-18

**Authors:** Janek Vilisaar, Kiyokazu Kawabe, Manjit Braitch, Jehan Aram, Yasemin Furtun, Angela J. Fahey, Mark Chopra, Radu Tanasescu, Patrick J. Tighe, Bruno Gran, Charalabos Pothoulakis, Cris S. Constantinescu

**Affiliations:** 1 Division of Clinical Neurology, University of Nottingham, Nottingham, UK; 2 Department of Neurology, Carol Davila University of Medicine and Pharmacy, The Colentina Hospital, Bucharest, Romania; 3 Division of Immunology, University of Nottingham, Nottingham, UK; 4 Inflammatory Bowel Disease Center, Divison of Digestive Diseases, University of California at Los Angeles, Los Angeles, CA USA; 5 Division of Clinical Neuroscience, Clinical Neurology Research Group, University of Nottingham, Queen’s Medical Centre, South Block C Floor, Nottingham, NG7 2UH UK

**Keywords:** Substance P, Neurokinin-1 receptor, Human, IL-12, IL-23, Tachykinins, Human, PBMC

## Abstract

The neuropeptide substance P (SP) exhibits cytokine-like properties and exerts different effects in autoimmune inflammation. Various immune cells express SP and its neurokinin-1 receptor (NK1R) isoforms. A role for SP has been demonstrated in a number of autoimmune conditions, including multiple sclerosis (MS). In this work, we studied the role of SP and NK1R in human immune cells with a focus on their relationship with IL-12/IL-23 family cytokines and the associated IFN-γ/IL-17. AIMS: (1) To determine the role of SP mediated effects on induction of various inflammatory cytokines in peripheral blood mononuclear cells (PBMC); (2) to investigate the expression of SP and its receptor in T cells and the effects of stimulation with IL-12 and IL-23. Quantitative real-time PCR, flow cytometry, ELISA, promoter studies on PBMC and primary T cells from healthy volunteers, and Jurkat cell line. Treatment with SP significantly increased the expression of IL-12/IL-23 subunit p40, IL-23 p19 and IL-12 p35 mRNA in human PBMC. Expression of NK1R and SP in T cells was upregulated by IL-23 but a trend was observed with IL-12. The IL-23 effect likely involves IL-17 production that additionally mediates IL-23 effects. Mutual interactions exist with SP enhancing the cytokines IL-23 and IL-12, and SP and NK1R expression being differentially but potentially synergistically regulated by these cytokines. These findings suggest a proinflammatory role for SP in autoimmune inflammation. We propose a model whereby immunocyte derived SP stimulates Th1 and Th17 autoreactive cells migrating to the central nervous system (CNS), enhances their crossing the blood brain barrier and perpetuates inflammation in the CNS by being released from damaged nerves and activating both resident glia and infiltrating immune cells. SP may be a therapeutic target in MS.

**Electronic supplementary material:**

The online version of this article (doi:10.1007/s11481-015-9589-x) contains supplementary material, which is available to authorized users.

## Introduction

Substance P (SP) is an ubiquitously found 11-amino acid peptide with a number of biological functions. It belongs to the family of tachykinins, which also includes neurokinin A, neurokinin B, neuropeptide K, neuropeptide Y, and hemokinin-1. SP is generated through the enzymatic cleavage of pre-pro-tachykinin A, which is encoded by the TAC1 gene. Splice variants of TAC1 also generate neurokinin A and neuropeptide K. The role of SP in pain pathways and neurogenic inflammation has been well established, including effects of vasodilatation and plasma extravasation. With its effects on smooth muscle, SP is known to be involved in motility of different organ tracts as well as stimulation of glandular secretion and modulating autonomic reflexes.

SP originates from several cellular sources. It is released by peripheral nerve endings and central terminals of sensory neurons in the central nervous system (CNS). In addition, it is produced by a variety of cells of the immune system. This, and the presence of its receptor, the neurokinin-1 receptor (NK1R) on cells of the immune system, suggest a role for SP in immune responses. Immunomodulatory properties and its role in autoimmune inflammation have only recently been investigated in more detail. A role for SP has been demonstrated in neuroimmune and systemic autoimmune/inflammatory conditions, including multiple sclerosis (MS), rheumatoid arthritis and inflammatory bowel disease (Goode et al. 2000; Kostyk et al. 1989; Lotz et al. 1987; O’Connor et al. 2004; Weinstock 2014), as well as in infections and cancer.

NK1R is a widely expressed G-protein coupled receptor, which can be expressed as a full length receptor in neuronal and non-neuronal cells, and a recently studied truncated isoform (NK1R-T), expressed in monocytes and other peripheral cells (Douglas and Leeman 2011). There is evidence that the proportion of the full length and truncated receptor varies by cell type and in disease states, such as inflammation and cancer (Chernova et al. 2009; Douglas and Leeman 2011).

In this article we discuss the interactions of SP and NK1R with the IL-12/IL-23 family of inflammatory cytokines with emphasis on the role of these molecules in neuroinflammatory conditions, including MS and its animal model, experimental autoimmune encephalomyelitis (EAE).

SP and NK1R are involved in neuroimmune communication, being abundantly expressed in the nervous system and its sensory nerve projections into peripheral immune organs, and immune cells themselves (lymphocytes, cells of monocyte lineage, dendritic cells, eosinophils, mast cells etc.). SP is involved in both innate and adaptive immune responses. The functions of SP involve induction of cytokines (see below), activation of immune cells (Nio et al. 1993; Scicchitano et al. 1988), modulation of T helper phenotype commitment (Brogden et al. 2005; Cunin et al. 2011; Levite 1998), chemotaxis (Ahluwalia et al. 1998; Ruff et al. 1985; Schratzberger et al. 1997), expression of adhesion molecules and effects on the blood–brain barrier (Matis et al. 1990; Vishwanath and Mukherjee 1996). A number of these effects have been linked to NK1R dependent activation of NFκB.

The effects include stimulation of the production of IL-1, IL-6, IL-8, IL-10, TNF-α from monocytes and macrophages (Ho et al. 1996; Kincy-Cain and Bost 1997; Laurenzi et al. 1990; Lotz et al. 1988), and IL-1, IL-2, IFN-γ, IL-4, IL-6, TNF-α and IL-10 from T cells (Blum et al. 1993; Calvo et al. 1992; Delgado et al. 2003).

Induction of IL-12 by SP has been demonstrated in the murine immune system (Kincy-Cain and Bost 1997) but until now not in the human immune system. IL-23 induction by SP has been reported in human monocytes (Cunin et al. 2011). In murine models, IL-12 has been shown to induce SP precursor mRNA in macrophages via STAT4 pathway (Arsenescu et al. 2005) and NK1R expression by both IL-12 and IL-18 stimulation via NFκB in T cells (Weinstock et al. 2003). Recently, IL-12 and IL-23 have been found to induce SP synthesis in murine T cells and macrophages, which can be regulated by IL-10 and TGF-β respectively (Blum et al. 2008).

EAE is a widely used model of MS with which it shares pathological, immunological, neurobiological, and clinical similarities (‘tHart et al. 2011; Constantinescu et al. 2011).

IL-12-induced IFN-γ production is the hallmark of the Th1 immune responses, whereas IL-17 is the signature cytokine of the Th17 proinflammatory pathway. Of note, studies showing SP induction of IFN-γ suggest that direct stimulation of NK1R is sufficent and the process does not require IL-12 (Blum et al. 2003). Moreover, although IL-23 is still thought to be an important cytokine for the stimulation and maintenance of the Th17 pathway, in particular in the human immune system, studies have shown that TGF-β in a proinflammatory environment that includes IL-6 and possibly IL-1, is responsible for Th17 differentiation (Bettelli et al. 2006). Although some discrepancies are still unresolved (Jagessar et al. 2012; Sanvito et al. 2010), a great number of studies are congruent in indicating a role for both Th1 and Th17 cells in MS and EAE (Edwards et al. 2010).

Studies in EAE have shown suppression of the disease in NK1R knockout mice and amelioration of T cell transfer EAE by NK1R antagonist CP-96345 (Nessler et al. 2006; Reinke et al. 2006). Additionally, the role for SP in the pathogenesis of MS has been suggested by genome-wide linkage studies highlighting SP precursor protein encoding TAC1 gene as a possible susceptibility gene for MS (Cunningham et al. 2005; Vandenbroeck et al. 2002).

In this work, we investigate the reciprocal regulation of substance P and NK1R and the IL-12/23 family with their associated inflammatory cytokines in the human immune system. Our findings show many similarities with those seen in a murine immune system, with relvance to many inflammatory diseases. We offer a perspective of how these interactions place SP as an important mediator of neuroinflammatory disease.

## Methods

### Cell Preparations and Cultures

PBMC were isolated from whole blood by standard density gradient centrifugation. For T cell activation, PBMC were cultured for 72 h with 10 μg/ml phytohemagglutinin (PHA-P, Sigma-Aldrich) in 10 % FCS/RPMI media. After media change, cells were stimulated with 1000 U/ml IL-2 (PeproTech) at 37 °C for 24 h. The resulting cells “blasts” are typically >95 % CD3+.

CD4+ cells were magnetically isolated using the human CD4+ T cell Isolation Kit (Miltenyi Biotec, USA), following the manufacturer’s protocol. These cells were then transferred into a 24-well plate pre-coated overnight with 0.5 ml/well of a mixture of anti-CD3 (0.5 μg/ml) (BD Pharmingen) and anti-CD28 (2.5 μg/ml) (BD Pharmingen) in PBS. The coated wells were washed with 1 ml RPMI medium prior to CD4+ cells transfer (10^6^ cells/well).

The Jurkat T cell line (kindly donated by Professor David Heery, School of Pharmacy, University of Nottingham, UK) was used for NK1R promoter-reporter assay.

### Stimulation of Cell Cultures

PBMC 10^6^/ml were stimulated for 24 and 48 h with 10^−6^–10^−12^ M SP (Sigma-Aldrich) with and without the presence of NK1R antagonist CP-96345 (Pfizer) 10^−5^–10^−6^ M, its inactive enantiomer CP-96344 (Pfizer) 10^−5^–10^−6^ M, anti-NK1R antibody (Chemicon International Inc) 1:500 or 1:1000, lipopolysaccharide (LPS, E. coli serotype 0111:B4, Sigma-Aldrich) 1 μg/ml. After stimulation, the supernatants were removed and stored at -80 °C for ELISA. Stimulations with cytokines were carried out at the following final concentrations: 10 ng/ml IL-12 (R&D Systems) with and without 2.5 μg/ml anti-IFN-γ antibody (PeproTech EC), 10 ng/ml IL-23 (R&D Systems) with and without 2.5 μg/ml anti-IL-17 antibody (PeproTech), 10 ng/ml IL-17 (PeproTech), 10 ng/ml IFN-γ (PeproTech) for 8 and 24 h for mRNA-level, and 24 and 48 h for protein-level expression.

### Flow Cytometry

Flow cytometry was performed for protein level expression of NK1R in PHA-stimulated PBMC (T cell blasts). After overnight incubation, stimulated T cell blasts were transferred into FACS tubes and 20 μl EDTA 100 mM (Sigma-Aldrich) were added to each tube and incubated for 5 min at room temperature, the cells were then fixed using formaldehyde 4 % (1 ml/tube) and left for 5–10 min at room temperature prior to washing three times, each with 1 ml PBA (Phosphate Buffered Albumin, containing 0.5 % bovine serum albumin in phosphate buffered saline), stained with 2.5 μl of monoclonal surface antibodies (anti-CD3-ECD, clone UCHT1, Beckman Coulter; anti-CD8-PeCy7, clone SFCI21Thy2D3, Beckman Coulter; and anti-CD56-PE, clone HCD56, Biolegend, and isotype control-PE, clone P3.6.2.8.1, eBioscience) and incubated for 40 min in the dark at 4 °C. The cells were then washed once with PBA and twice, each with 1 ml perm/wash buffer (BD Biosciences), stained with 10 μl anti-NK1R-APC antibody (R&D Systems, Clone 694501) and its isotype control (mouse IgG3-APC, clone 133316, R&D Systems) and incubated in the dark at room temperature for 30 min. Cells were washed once with perm/wash buffer, fixed with 0.4 ml of 0.5 % formaldehyde and analysed.

Regarding CD4+ cells staining, 20 μl EDTA (100 mM) and 1 ml formaldehyde 4 % were added as above. The cells were washed three times with 1 ml perm/wash buffer and 10 μl anti-NK1R-APC antibody were added. The cells were incubated for 30 min in the dark at room temperature, washed once with 1 ml perm/wash buffer, and fixed with 0.4 ml formaldehyde 0.5 %. For flow cytometry experiments, FC500 (Beckman Coulter) flow cytometer and Weasel 3.1 software were used. For data presentation, GraphPad Prism 6.0 software was used. The quadrants were set in flow cytometry plots using single colour control tubes, fluorescence minus one (FMO) tubes, and isotype control tubes.

### ELISA

Commercial kits were used for measuring concentrations of IL-1β (R&D Systems) and the different IL-12 and IL-23 subunits in stimulated PBMC culture supernatants, as well as SP in T blast supernatants. IL-12p40 (Diaclone), IL-12p70 (eBioscience) and IL-23 p19/p40 (eBioscience) kits were used following manufacturer’s instructions with assay sensitivities specified as follows: below 2.58 pg/ml for IL-1β, below 20 pg/ml for IL-12p40, below 15 pg/ml for IL-23 p19/p40 and 4 pg/ml for IL-12p70. For measuring SP in T blast supernatants, Parameter™ SP ELISA kit (R&D Systems) was used according to the instructions with 31.5 pg/ml as the mean minimum detectable concentration of SP.

### Quantitative Real-Time Polymerase Chain Reaction (PCR)

Quantitative real-time reverse transcriptase PCR was used to assess mRNA abundance of IL-12, IL-23 subunits in PBMC on stimulation with SP, and mRNA abundance of SP precursor and NK1R isoforms in PHA/IL-2 T blasts that had been stimulated with IL-12, IL-23, IL-17, IFN-γ. Total-RNA was extracted by column extraction using RNeasy Miniprep Kit® (Qiagen) following the manufacturer’s instructions: the homogenization step was performed by needle homogenization. First strand cDNA synthesis was initiated from 0.5 μg total RNA using random primers (Promega, USA) and MMLV reverse transcriptase (Promega) using conditions specified by the manufacturer in a final volume of 25 μl. The following specific primer sequences were used for IL-23p19 sense: 5′ctccctgatagccctgtg3′, antisense: 5′gactgaggcttggaatct3′; for IL-12p40 sense: 5′ggagtaccctgacacctg3′, antisense: 5′agatgaccgtggctgagg3′; for IL-12p35 sense: 5′ccactccagacccaggaatg3′, antisense: 5′gacggccctcagcaggt3′; for NK1R-F (full length): sense 5′gaatgaggacagtgacgaac3′, antisense 5′ttgtggaacttgcagtagaac3′; for NK1R-T (truncated): sense 5′tcttcttcctcctgccctacatc3′; antisense 5′tggagagctcatggggttggatcct3′; for TAC1: sense 5′tcaatgggcaatgacaggta3′, antisense 5′tccgcagtagctgacacaac3′. As an internal standard, β2-microglobulin (β2MG) with the following primers was used: sense 5′ctccgtggccttagctgtg3′, antisense 5′tttggagtacgctggatagcct3′ (primers from MWG Biotech, Germany). For primer design Primer 3 software was employed. Real-time PCR reactions were carried out using the SYBR Green fluorescence method with SYBR Green Mastermix (Stratagene) as specified by the manufacturer. All real-time PCR reactions were carried out in triplicate on multiplex qPCR system Mx4000® (Stratagene) using default thermal cycling conditions. Dissociation curves were included routinely for each reaction for assessing product homogeneity. For quantitation of transcripts, the relative standard curve method, described by Applied Biosystems (AppliedBiosystems 1997), was used. For the set of standards, equal aliquots of undiluted cDNA from each sample were pooled together and serially diluted (neat, 1:2, 1:5, 1:10, 1:20). Using the standard curve, the comparative threshold cycle value was converted to nanograms of total RNA equivalent, used for first strand synthesis for both the target gene transcript and the housekeeping gene, β2MG. Subsequently, the values of individual sample mRNA abundance for the target gene were normalized with the corresponding values of β2MG. The reaction efficiency was optimal with template in 1:5 dilution and primer concentrations 10 pmol/μl; for TAC1 reaction the concentration of primers 2 pmol/μl and 1:5 template dilutions were used for optimal efficiency.

### Transfection of Jurkat Cells With NK1R Promoter-Reporter Construct and Luciferase Reporter Assay

The vector for the NK1R promoter activation assay was prepared by transforming E coli DH5α –T1 competent cells (Invitrogen) with human NK1R gene promoter 1,837-bp fragment, cloned into luciferase expression vector pGL3-Basic (Promega) . For incorporation of the vector into Jurkat cells, 2 × 10^7^ cells/400 μl ice-cold PBS were transferred into each electroporation cuvette (Invitrogen) and kept on ice for 10 mins. Subsequently, 3 μg prepared NK1R promoter-reporter plasmid (containing *Photinus pyralis* luciferase) and 0.3 μg *Renilla reniformis* luciferase reporter vector (Promega) as an incorporation control were added per cuvette and pulsed on Gene Pulser X cell™ (Biorad) with 250 V and 950 μF. After electroporation, cells were kept in 10 % FCS/RPMI overnight at 37 °C, 5 % CO_2_. Following stimulation of transfected cells with cytokines as above for 24 h, cells were washed with PBS and passively lysed according to the Dual-Luciferase Reporter Assay protocol (Promega), whilst kept on a shaker for 15 min. The lucifearase activities of each lysate were sequentially read on TD-20/20 single-sample luminometer (Turner Designs) by adding initiation/stop reagents (Promega) per protocol. Background luminescence of non-transfected control was subtracted from individual sample values and ratios of *Photinus*/*Renilla* luciferase signals were calculated.

### Statistical Analysis

Data were analysed using nonparametric tests: Friedman test for comparisons of means of ordinal data, or Wilcoxon test for continuous variables. SPSS 16.0 software was employed for analysis. Differences were considered statistically significant when *p* was ≤0.05.

## Results

### Protein-Level Expression of IL-1β and IL-12p40 in PBMC Stimulated with SP

PBMC (10^6^/ml) were stimulated with SP 10^−6^, 10^−9^ and 10^−12^ M, and IL-1β and IL-12p40 were measured by ELISA after 36–48 h. SP alone was unable to induce detectable IL-1β, unless co-stimulation with bacterial lipopolysaccharide (LPS) was used (data not shown). SP 10^−6^ M enhanced IL-12p40 from 53 (±36) pg/ml to 135 (±2) pg/ml (*p* = 0.02). Lower concentrations of SP failed to induce IL-12p40 in the absence of co-stimulation with LPS (data not shown). Because the amounts of IL-12p40 are likely to be too low to correspond to detectable levels of IL-12p70 or IL-23 protein by ELISA, we measured the mRNA expression of the IL-12 family cytokines.

### mRNA-Level Expression of IL-12 and IL-23 Subunits in PBMC on Stimulation with SP

**Fig. 1:**
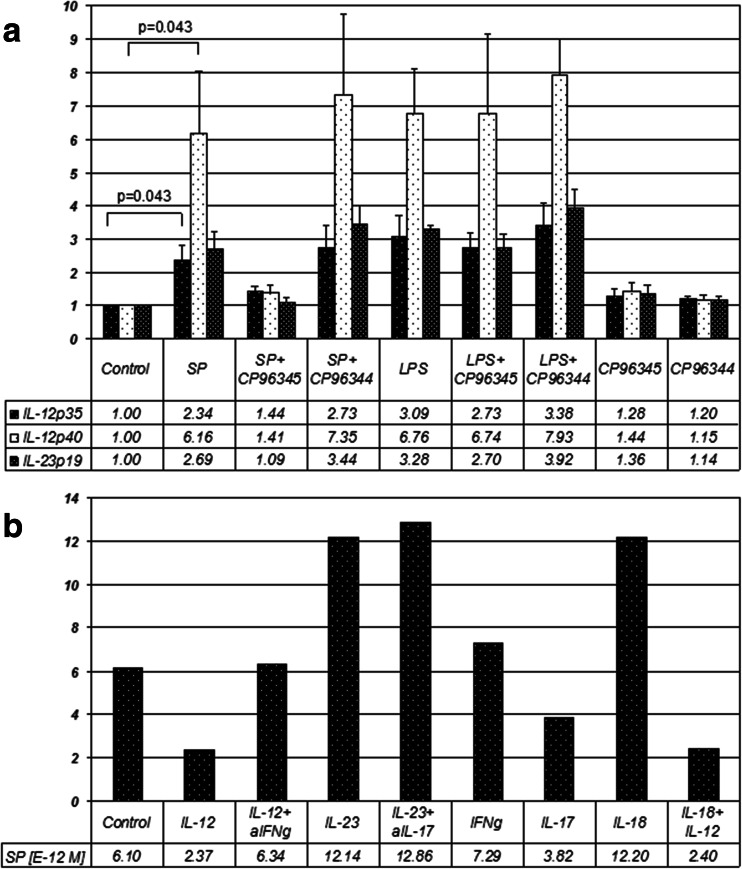
**a** Quantitative PCR results showing IL-12p35, IL-12/23p40 and IL-23p19 mRNA abundance in PBMCs in response to 24 h incubation with different stimuli. The values represent arbitrary units after normalization with corresponding values of β2MG as an internal standard. Each bar represents averages of 5 different healthy donors, error bars indicate SEM; **b** representative results from one healthy donor showing molar concentrations of SP (times 10^−12^ M) in cell culture supernatants on ELISA, following a 24 h stimulation of T blasts with different stimuli. The concentrations are converted from mass concentrations (pg/ml) given on ELISA

To assess IL-12 and IL-23 induction by SP in PBMC, experiments with a 24-h induction of the IL-12/23p40, IL-12p35 and IL-23p19 subunit mRNA were done on 5 healthy volunteers. The expression of the three IL-12 and IL-23 subunits showed a similar response to stimulation with SP and suppression by CP-96345 (specific NK1R antagonist), but not by CP-96344 (control with no antagonist properties). SP strongly upregulated the expression of the subunits to a degree similar to LPS (Fig. 1a). The induction of the p40 subunit by SP was greatest with a 6.2-fold increase (*p* = 0.043), followed by p19 and p35 induction with a 2.7-fold (*p* = 0.068) and 2.3-fold (*p* = 0.043) increase in their expression respectively.

The product homogeneity and size for each of the conditions was confirmed on agarose gel electrophoresis with a product size of 131 bp for IL-12/23p40, 62 bp for IL-12p35, 109 bp for IL-23p19 and 148 bp for β2MG.

### Expression of NK1R and SP in Lymphocyte Populations and Effects of IL-12 and IL-23

The expression of the NK1R receptor on subgroups of human immune cells is not well known. We determined this expression by flow cytometry using a directly conjugated antibody. The percentages of NK1R+ cells in PHA-stimulated T cells (“blasts”), and subgroups of blasts based on expression of cell surface markers CD3, CD8, and CD56, unstimulated or stimulated with IL-12 or IL-23 for 24 h, and gated for CD3 (CD3-PC7, NK1R-PE) can be found in supplemental Table 1 online. In addition, the percentages of NK1R+ cells among the magnetically separated CD4+ T cells grown on anti-CD3/anti-CD28-coated plates and stimulated as above are shown in the table.

Both IL-12 and IL-23 appeared to modestly (not statistically significantly) increase NK1R in PHA-stimulated T cells (“blasts”), and subgroups expressing CD3, CD8, and CD56 (Table 1 and Supplemental Figure 1 Online). Similar slight but not statistically significant trends towards increased NK1R expression were observed with 24 and 48 h stimulation with IL-12 plus IL-18, IL-18, IL-23, IL-17 and IFN-γ (not shown).

For SP peptide induction, two separate ELISAs were run on supernatants of stimulated T cells from 5 control subjects (Fig. 1b). Peptide level SP expression showed a great deal of variability between subjects. None of the stimulation conditions studied, i.e. IL-12, IL-12 plus anti-IFN-γ, IL-23, IL-23 plus anti-IL-17, IL-18, or IL-12 plus IL-18 showed effects that were statistically significant at the protein level.

### mRNA Level Induction of NK1R and SP in T Cells

The results shown so far, regarding NK1R, refer to the full-length transcript. We have examined the NK1R isoform expression in T cell blasts with similar expression of the truncated (NK1R-T) versus full-length NK1R (NK1R-F; “NK1R” in this article refers to NK1R-F) isoform in unstimulated T cell blasts (ratio of 1.13; 53 % versus 47 %, respectively, *p* = 0.95).

**Fig. 2:**
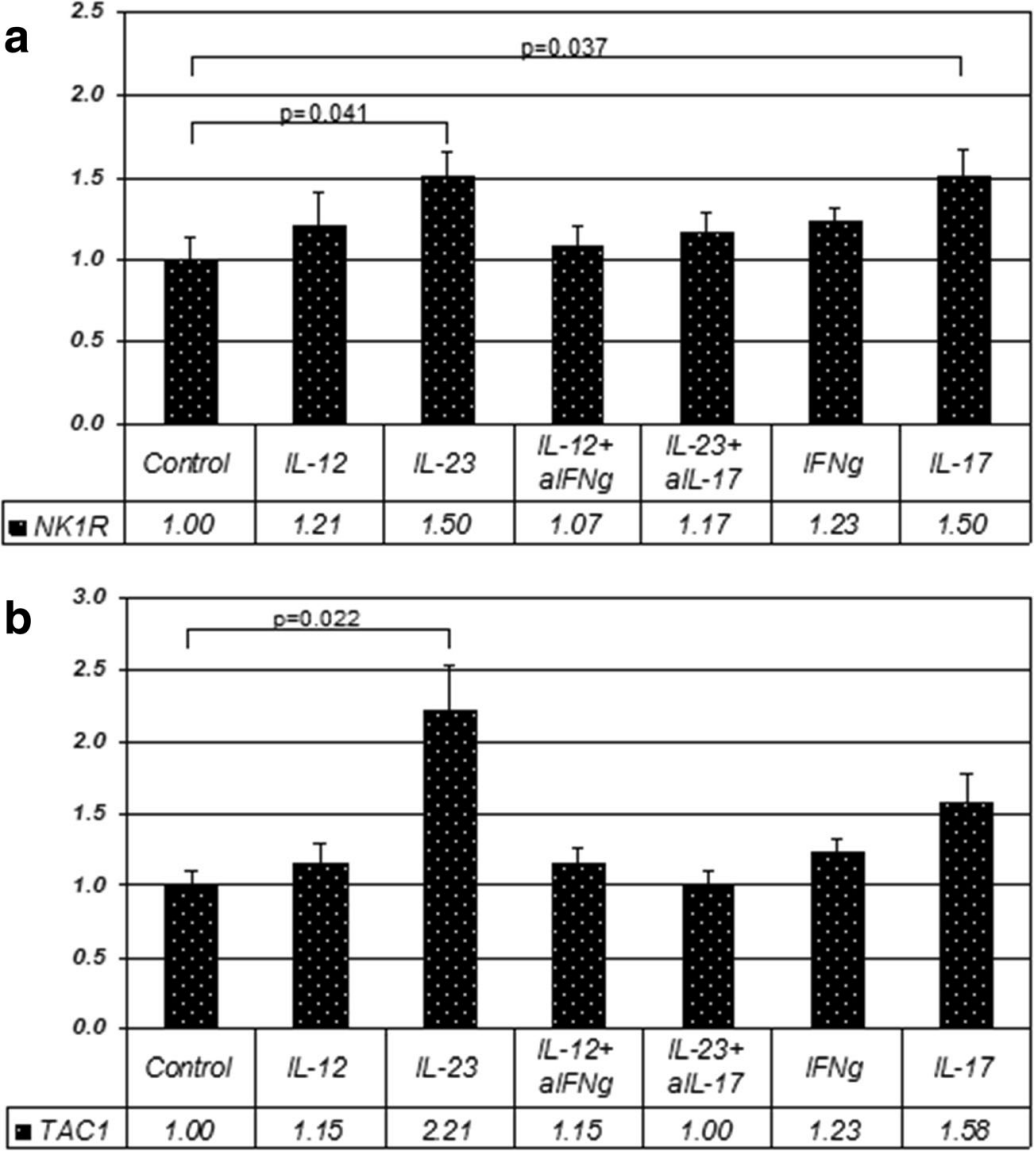
**a** Quantitative PCR was used to assess mRNA-level expression of NK1R relative to internal standard β2MG. Each *bar* represents an average of 10 stimulation assays on T cell blasts from 10 different healthy donors +/- SEM. The results of individual assays were expressed in arbitrary units of mRNA abundance, normalised with the corresponding values of β2MG; **b** mRNA-abundance of TAC1 relative to internal standard β2MG: mean ratios of stimulation assays on T blasts from 10 different healthy donors +/- SEM

The effects of different cytokine stimuli on SP precursor and NK1R mRNA level expression were studied in PHA/IL-2-stimulated T cell blasts from 10 healthy volunteers. IL-23 significantly upregulated both NK1R (*p* = 0.041) and TAC1 transcripts (*p* = 0.022) (Fig. 2a and b). There was a trend to upregulation of TAC1 and NK1R by IL-12 (Fig. 2). Therefore, we restricted the NK1R-T analysis to the IL-23 stimulation. We observed a preferential induction of NK1R-T upon stimulation with IL-23 compared to NK1R-F (ratio 4:1; *p* = 0.007).

NK1R (*p* = 0.037) but not TAC1 (*p* = 0.203) expression was also significantly upregulated by IL-17A (here referred to as IL-17). Anti-IL-17 abolished the effects of IL-23 in inducing TAC1 (*p* = 0.041), but not NK1R (*p* = 0.262) (Fig. 2). On the other hand, anti-IFN-γ did not block IL-12 effects in these experiments.

The above effects were studied over different stimulation periods as IL-12 and IL-23 responses may prevail at different stages of inflammation (Thakker et al. 2007). Stimulation for 8 and 24 h was used, as possible induction of secondary cytokines to IL-12 and IL-23 stimuli may take time to initiate, however, no differences were found in T cell blasts between 8 and 24 h. The results from a 4 h-stimulation as used by Weinstock et al. (Weinstock et al. 2003) and Arsenescu et al. (Arsenescu et al. 2005) were also not significantly different from the effects at 8 and 24 h.

**Fig. 3:**
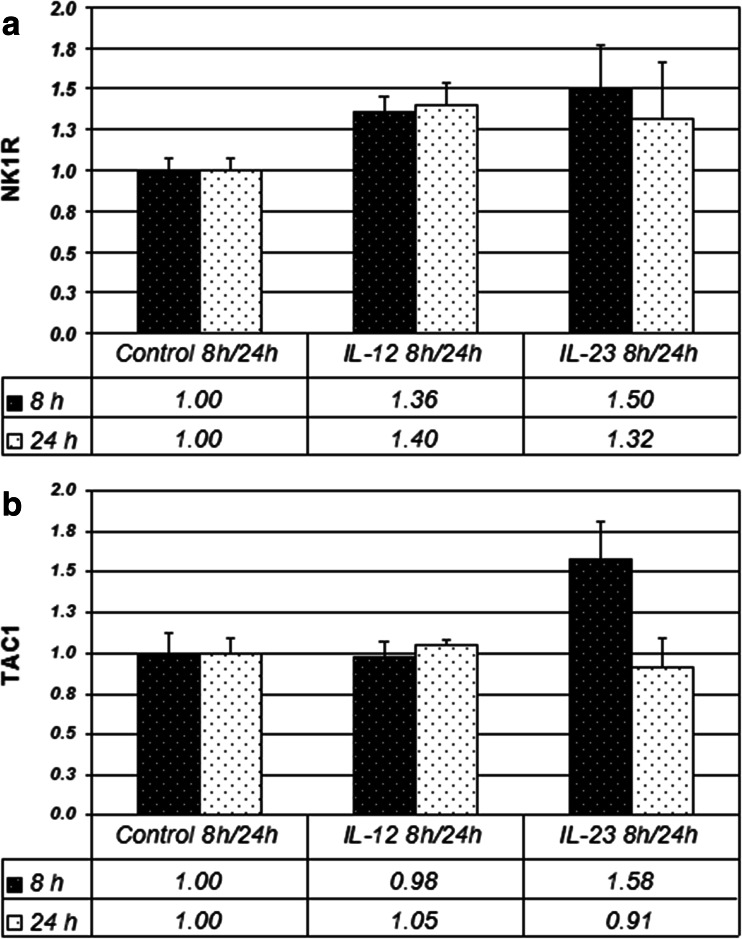
NK1R and TAC1 mRNA abundance in CD4+ cells following 8 and 24 h stimulation (10 ng/ml IL-12 or 10 ng/ml IL-23), normalized with β2MG as internal standard. The values represent mean ratios of 4 healthy donors +/- SEM

However, in the experiments done on CD4+ cells, a slightly greater tendency for NK1R induction by IL-12 was seen at 24 h as compared to 8 h, and in SP induction by IL-23 at 8 h as opposed to 24 h (Fig. 3). With the 8 and 24 h sets combined, NK1R induction by IL-23 in CD4+ cells was significant (*p* = 0.043), and induction by IL-12 showed only a trend (*p* = 0.063).

### NK1R Promoter Construct Activation in Jurkat Cells

IL-23 appears to be a potent inducer of the NK1R promoter region with an average 6-fold increase in the reporter signal ratio compared to the unstimulated cells (Supplemental Figure 2 online). In both of these experiments, anti-IL-17 in IL-23 stimulated samples showed strong reduction of NK1R expression, compared to the IL-23 stimulus alone. IL-12 and IFN-γ showed little activation in these assays. IL-17, IL-18 and the combination of IL-12 and IL-18 showed moderate effects with 2.1, 2.3 and 2.8-fold average increase from the baseline respectively.

## Discussion

While evidence exists for a potential role of SP in experimental and human autoimmune neuroinflammatory disease, and the role of the IL-12 family and related inflammatory cytokines is convincing, little is known to date about the induction of the IL-12 family cytokines by SP in human immune cells or about the effects of proinflammatory cytokines on the expression of NK1R in human immune cells (Weinstock 2014). In animal models, SP induced or stimulated Th1 type and Th17 immune responses (Beinborn et al. 2010), or inhibited anti-inflammatory cytokines as recently shown in murine dendritic cells (Janelsins et al. 2013).

In our study on human PBMC, a small degree of induction of IL-12p40 by SP was observed at the protein level. The small amounts of IL-12p40 suggested that sensitivity of the ELISA would be too low to detect the heterodimeric cytokines IL-12p70 and IL-23. The sensitivity of the Cytometric Bead Array (CBA), lower than the individual ELISA, had been also tested before ELISA measurements and found insufficient to detect the heterodimeric cytokines in our series (data not shown). Small effects of SP on IL-12 secretion are consistent with the published report (Janelsins et al. 2013).

When studying mRNA-level expression of the individual subunits, induction of all subunits is detectable. The highest values were seen with p40, consistent with the role of p40 as part of both IL-12 and IL-23. The common p40 may also be found in free monomer and in homodimers form in the mouse immune system. The mRNA abundance of both IL-12p35 and IL-23p19 was lower than that of p40, whereas the abundance of p19 was slightly higher than that of p35. The results suggest potentially distinct molecular mechanisms for IL-12 and IL-23 induction by SP, possibly involving distinct signalling pathways and downstream regulatory factors (Goodridge et al. 2003) at translational level or post-translational processing level as known for p35 (Murphy et al. 2000).

IL-12 induction by SP has been previously shown at mRNA level in murine macrophages (Kincy-Cain and Bost 1997). In that study, SP agonist concentrations as low as 10^−9^ M were bioactive, while 10^−6^ M had an effect in our study. In the study by Kincy-Cain, both IL-12p40 and IL-12p35 mRNA were potently induced; however, an increase in IL-12p70 expression was not detected. At the protein level at 24 h, IL-12p40 was induced, however, no detectable secretion of p70 occurred (Kincy-Cain and Bost 1997). We have not tested p70 in our study. Additive effects of SP on LPS in IL-12 induction were observed as were also seen in our experiments on PBMC.

IL-23 upregulates both NK1R and SP precursor mRNA and appears to be a stronger stimulus than IL-12 in human T cells. Whereas IL-12 can act on both naïve and activated cells, mainly activated cells are considered responsive to IL-23 (Parham et al. 2002).

In protein-level experiments, SP did not show consistent induction by the cytokines used. However, too much significance cannot be put on detected SP concentrations in cell culture supernatants as SP is quickly inactivated in aqueous solutions near physiological pH. The discrepancies between the protein and mRNA level induction will be discussed further below.

There was more pronounced inter-individual variability in TAC1 than in NK1R expression. This is expected as there are more factors known in TAC1 transcriptional and post-transcriptional regulation, providing a source for variation. These factors include transcriptional silencers repressing TAC1 transcription in non-neuronal cells (Greco et al. 2007). Within exon 1 in TAC1 promoter region, adjacent to NFκB site, two RE-1 silencer of transcription (REST) binding sites have been identified (Greco et al. 2007). Additionally, RNA-binding proteins and microRNAs regulate SP synthesis (Greco and Rameshwar 2007; Murthy et al. 2008). Although TAC1 mRNA is found ubiquitously in human non-neuronal tissues (Pinto et al. 2004), some studies suggest the importance of preconditioning non-neuronal cells with inflammatory stimuli for TAC1 expression. For example, IL-1α has been shown to induce TAC1 and particularly SP translation in non-neuronal cells (Greco and Rameshwar 2007).

Interestingly, we show that IL-23 stimulation increases the proportion of NK1R-T transcript in T cells. This truncated receptor has been implicated in the mechanisms whereby chronic inflammation transits to malignancy, a mechanism in which the IL-23/IL-17 pathways seems to play a role (Chernova et al. 2009).

Both NK1R and the TAC1 promoter regions have NFκB binding sites (Simeonidis et al. 2003; Takahashi et al. 1992). Here we have shown that NK1R promoter is activated by IL-23 and, as suggested by anti-IL-17 blocking effects, also by IL-17. Both IL-23 and IL-17 are known to signal via NFκB, consistent with our findings. Additionally, IL-18 showed similar moderate effects as IL-17 on the promoter level. In the work by Weinstock and colleagues (Weinstock et al. 2003) and Arsenescu and colleagues (Arsenescu et al. 2005), NK1R expression was induced by IL-12 and IL-18 in murine T cells via NFκB, whereas SP induction in murine macrophages was responsive only to IL-12 via STAT4. In our study, the effects of IL-18 and co-stimulation of IL-12 and IL-18, activating NK1R promoter, were less than the effects of IL-23. IL-12 alone showed no activation of NFκB-containing NK1R promoter, and it is known to act via STAT4. SP synthesis has been previously shown to be STAT4 independent (Blum et al. 2008).

IL-12 has previously been shown to induce SP which can mediate IFN-γ production, potentiating IL-12 effects in murine cells (Blum et al. 1993, 2001). Thus, SP has been so far suggested to be part of the Th1 pathway. In a more recent study, IL-12 was shown to induce SP in murine T cells, whereas IL-23 induced SP in macrophages (Blum et al. 2008). In our study, we also show evidence for SP pro-inflammatory effects on IL-23 and IL-17 pathways in humans, and a prominent effect of IL-17A and IL-23 on NK1R. Although less prominent, the effects of IL-12/IFN-γ and their interaction with SP and NK1R seem to follow the same directions as those seen in the murine immune system and to indicate a synergistic effect to the IL-23/IL-17 cytokines. In the stimulation of NK1R for induction of IFN-γ, IL-12 may be in part redundant, as it has been shown that NK1R ligation by SP directly induces IFN-γ.

The role of SP can be best summarized as of a pleiotropic immune regulator. SP signalling involves activation of NFκB, which underlies the regulation of various inflammatory genes, explaining SP effects on chemotaxis and other inflammatory mechanisms. One of the important mechanisms is SP participation in regulating cytokine pathways.

There are important proinflammatory cytokines such as IL-6 that may be even more important for the development of Th17 responses than IL-23. It is known that SP induces IL-6 (Blum et al. 1993; Calvo et al. 1992; Delgado et al. 2003). Our preliminary multiple cytokine assays performed by cytometric bead arrays (not shown) showed little or no induction of IL-6 by SP in PHA-stimulated PBMC. This paper focused primarily on the IL-12 family of heterodimeric cytokines, in particular since some of the IL-12-SP regulation reports preceded the discovery of IL-23.

SP modulation of immune activities is characterized by mutual regulation. As SP regulates a multitude of cytokines and its pathway is also regulated by inflammatory activity, the reciprocal interactions between SP and its receptor and the IL-12/IL-23 cytokines are unlikely to be specific to this family of cytokines but more likely to be part of a wider interactive network. Preconditioning of cells may be important for optimal SP effects, and similarly, priming of cells with SP can enhance cytokine effects. Protein-level effects largely depend on these multiple stimulatory conditions which are likely to be present and undergo dynamic changes in vivo. In particular in the context of inflammation, multiple inflammatory signals may synergise to produce a powerful inflammatory effect. The positive feedback loop between SP and proinflammatory cytokines may be essential for the augmentation of the inflammatory response and various sites. In addition, this interaction may play a role not only in augmenting, but also in maintaining inflammation, leading to a chronic inflammatory state, where recent evidence suggest the truncated NK1R (NK1R-T) may play a role.

## Role of SP and its Interaction With IL-12/IL-23 and Related Cytokines in Autoimmune Neuroinflammation

As pointed out in a recent review on SP (Weinstock 2014), the immunoregulatory effects of SP are wide, and this can mean different or even opposite effects in different infections or inflammatory diseases: a protective role in some and a detrimental role in others. Even in conditions with similar etiologies, the regulation of and by SP and NK1R can be different. In inflammatory bowel disease, for example, there is initial strong activation of innate immune responses, and SP and NK1R are involved in this process. Thus blocking them could be of benefit.

MS is an autoimmune disease mediated predominantly by T cells (of Th1, Th17, and GM-CSF producing variety) (Constantinescu and Gran 2014). Both innate and adaptive immune responses play an essential role in MS (Podda et al. 2013)..

**Fig. 4:**
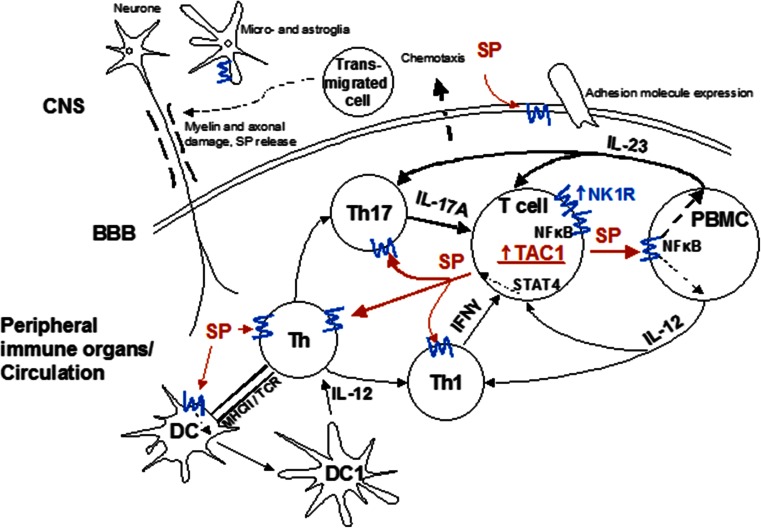
Schematic diagram illustrating substance P (SP) and cytokine interactions in autoimmune demyelinating disease (e.g. MS). For clarity, only the main effects on pro-inflammatory Th17 and Th1 pathways have been depicted with thicker arrows representing stronger responses suggested by our study. *Blue lines* indicate NK1R. SP effects are shown in *red. CNS* central nervous system; *BBB* blood–brain barrier; *DC* dendritic cell; *PBMC* peripheral blood mononuclear cell; *MHCII* major histocompatibility complex II; *TCR* T cell receptor

Where can the interaction of SP and NK1R with inflammatory cytokines investigated here occur in the autoimmune neuroinflammation of MS? In the prevailing model, autoreactive T cells become activated in the periphery through a variety of stimuli. SP plays an important role at this level, by enhancing proinflammatory cytokine production by these T cells, which can themselves produce more SP, up-regulate NK1R, and stimulate antigen presenting cells to produce further T cell stimulatory cytokines. This leads to an up-regulation of Th17 and Th1 responses, and enhanced penetration of the blood brain barrier (through up-regulation of NK1R and adhesion molecules on endothelial cells, among other mechanisms). In the CNS, SP stimulating cytokines, produced by infiltrating T cells and monocytes, and SP itself further stimulate both CNS-intrinsic cells with immune functions such as microglia and astrocytes. Consistent with this, SP-immunoreactive astrocytes are found in MS lesions (Kostyk et al. 1989), and CNS astrocyes and microglia express both IL-12 and IL-23 (Constantinescu et al. 2005). These processes facilitate perpetuation of inflammation, and a chronically activated microglia, as shown in progressive stages of MS (Lassmann et al. 2012) (Fig. 4).

A recently discovered mechanism may shed light on the complex role of TGF-β, which, in an inflammatory environment, induces Th17 responses. TGF-β reduces the otherwise rapid internalization of NK1R, not only increasing its availability to SP generated in CNS inflammation, with subsequent enhancement of IFN-γ and IL-17 production, but also leading to further Th17 differentiation of naive T cells (Bettelli et al. 2006).

The above proposed model indicates that blocking SP and its receptor can have a beneficial effect in autoimmune inflammation of the CNS such as occurs in MS, and that the effect may persist in the chronic stages of the condition. In the well-established MS model, EAE, the genetic absence of NK1R or its suppression using a synthetic antagonist was beneficial in the chronic stages of the disease (Reinke et al. 2006). In another study, a different antagonist also had therapeutic effects (Nessler et al. 2006). Notably, suppression of IFN-γ and of adhesion molecules were associated with the beneficial effects.

Given the similarity of the results presented here with those from the murine experiments, we conclude that SP, in part through its interaction with proinflammatory cytokines of the IL-12/IL-23, contributes to the development and perpetuation of autoimmune inflammation in the CNS, and it is a strong candidate as a therapeutic target in MS.

## Electronic supplementary material

Below is the link to the electronic supplementary material.

Supplemental Table 1 Expression of NK1R in various subsets of PBMCs from healthy human volunteers (n=3). The results are given as means and SEM of the percentage of cells expressing NK1R. (DOCX 13 kb)

Supplemental Figure 1 NK1R expression in: PHA/IL-2 stimulated PBMCs (“blasts”) (Histograms A-H) and magnetically isolated CD4+ lymphocytes (Histogram I). Cells Gating: (A), non-gated; (B), CD56+ cells; (C), CD3+CD8- lymphocytes; (D), CD3+CD8+CD56-lymphocytes; (E), CD3-CD56+ cells; (F), CD3+CD56+ lymphocytes; (G), CD3+ lymphocytes; (H), CD8+ lymphocytes; (I), Lymphocytes. Colour legend: Black: Unstimulated cells; Red: IL-12-stimulated; Blue: IL-23-stimulated; Pink: Isotype control-bound cells. NK1R-APC: Allophycocyanin-conjugated mouse monoclonal anti-human NK1R (GIF 299 kb)

High Resolution Image (TIFF 320 kb)

Supplemental Figure 2 Dual-luciferase reporter assay with activation of NK1R promoter-reporter construct in Jurkat cells. The results represent mean ratios of Photinus/Renilla luminescence of two experiments after 24 h stimulation with different conditions. (GIF 104 kb)

High Resolution Image (TIFF 36 kb)
