# Supplementary material for: Reciprocal Regulation of Substance P and IL-12/IL-23 and the Associated Cytokines, IFNγ/IL-17: A Perspective on the Relevance of This Interaction to Multiple Sclerosis
Source: J Neuroimmune Pharmacol. 2015 Feb 18;10(3):457–67. doi: 10.1007/s11481-015-9589-x (PMC4543419; doi:10.1007/s11481-015-9589-x)
Supplement: Supplementary file 1 — Expression of NK1R in various subsets of PBMCs from healthy human volunteers (n=3). The results are given as means and SEM of the percentage of cells expressing NK1R. (DOCX 13 kb) [file 11481_2015_9589_MOESM1_ESM.docx]

***Tables***

**Table 1.** Expression of NK1R in human lymphocytes

|  | **percentages of cells expressing NK1R+ (mean ± SEM)** | | |
| --- | --- | --- | --- |
| **Cells subtypes analysed** | **Unstimulated** | **IL-12-Stimulated** | **IL-23-Stimulated** |
| PBMCs blasts | 35.00 ± 2.84 | 38.73 ± 3.46 | 37.43 ± 3.03 |
| CD56+ cells in blasts | 33.47 ± 3.45 | 36.43 ± 6.78 | 38.47 ± 2.99 |
| CD3+ lymphocytes in blasts | 38.33 ± 1.88 | 41.73 ± 4.09 | 39.87 ± 1.32 |
| CD3+CD8- lymphocytes in blasts | 48.03 ± 0.92 | 50.53 ± 2.68 | 45.80 ± 1.32 |
| CD8+ lymphocytes in blasts | 46.23 ± 4.35 | 50.50 ± 5.58 | 51.70 ± 1.44 |
| CD3-CD56+ blasts | 40.20 ± 9.53 | 46.67 ± 6.89 | 32.53 ± 0.72 |
| CD3+CD56+ lymphocytes in blasts | 52.63 ± 3.68 | 56.20 ± 7.37 | 58.03 ± 2.08 |
| CD3+CD8+CD56- lymphocytes in blasts | 32.40 ± 2.62 | 34.67 ± 2.46 | 35.17 ± 1.94 |
| CD4+ lymphocytes magnetically isolated | 62.13 ± 9.27 | 55.07 ± 2.59 | 54.73 ± 5.10 |

Supplemental Table 1. Expression of NK1R in various subsets of PBMC healthy human volunteers (n=3). The results are given as means and SEM of the percentage of cells expressing NK1R.
