# Supplementary figures and images for: Reciprocal Regulation of Substance P and IL-12/IL-23 and the Associated Cytokines, IFNγ/IL-17: A Perspective on the Relevance of This Interaction to Multiple Sclerosis
Source: J Neuroimmune Pharmacol. 2015 Feb 18;10(3):457–67. doi: 10.1007/s11481-015-9589-x (PMC4543419; doi:10.1007/s11481-015-9589-x)

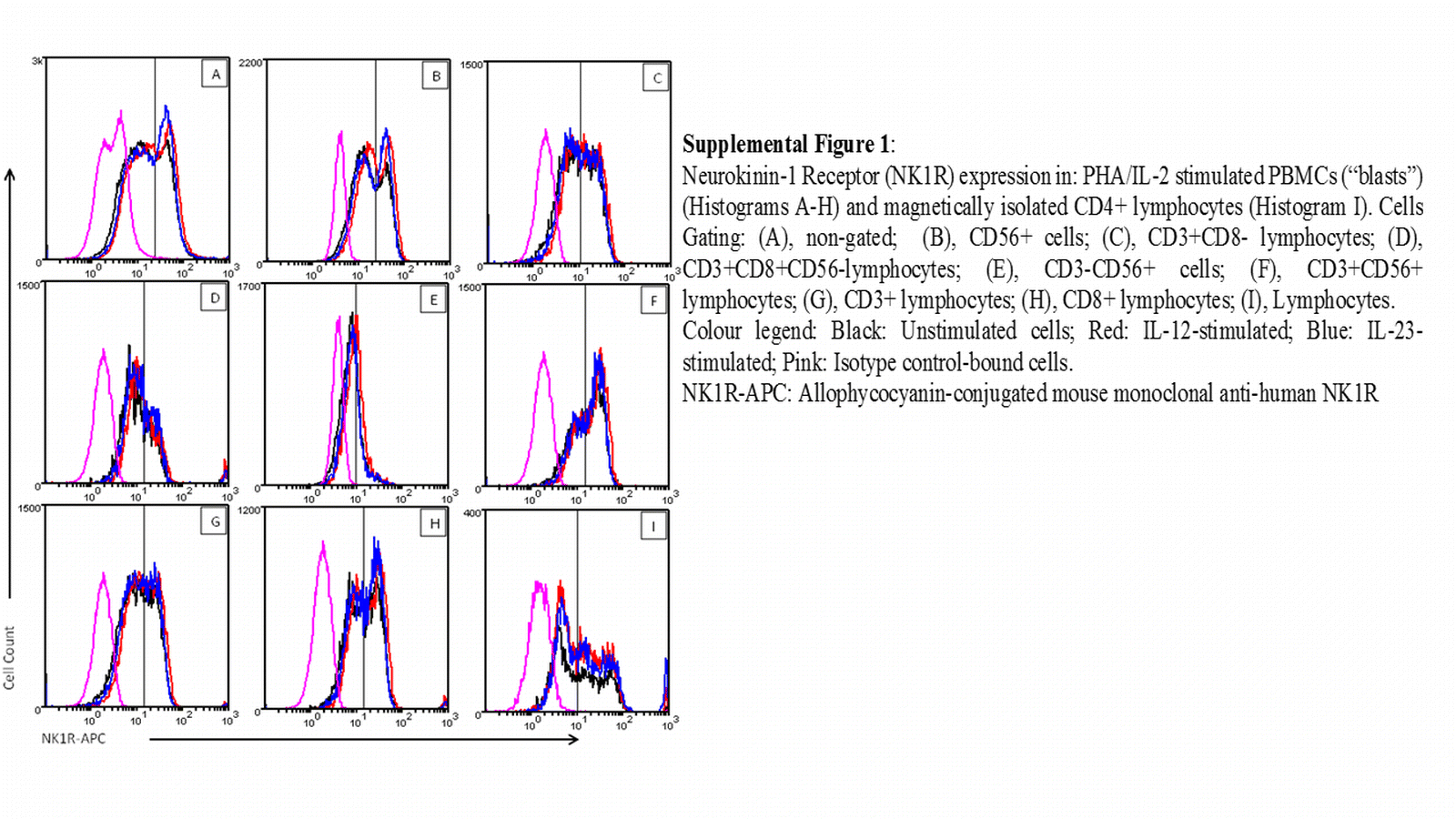

Supplement: Supplementary file 2 — NK1R expression in: PHA/IL-2 stimulated PBMCs (“blasts”) (Histograms A-H) and magnetically isolated CD4+ lymphocytes (Histogram I). Cells Gating: (A), non-gated; (B), CD56+ cells; (C), CD3+CD8- lymphocytes; (D), CD3+CD8+CD56-lymphocytes; (E), CD3-CD56+ cells; (F), CD3+CD56+ lymphocytes; (G), CD3+ lymphocytes; (H), CD8+ lymphocytes; (I), Lymphocytes. Colour legend: Black: Unstimulated cells; Red: IL-12-stimulated; Blue: IL-23-stimulated; Pink: Isotype control-bound cells. NK1R-APC: Allophycocyanin-conjugated mouse monoclonal anti-human NK1R (GIF 299 kb) [file 11481_2015_9589_Fig5_ESM.gif]

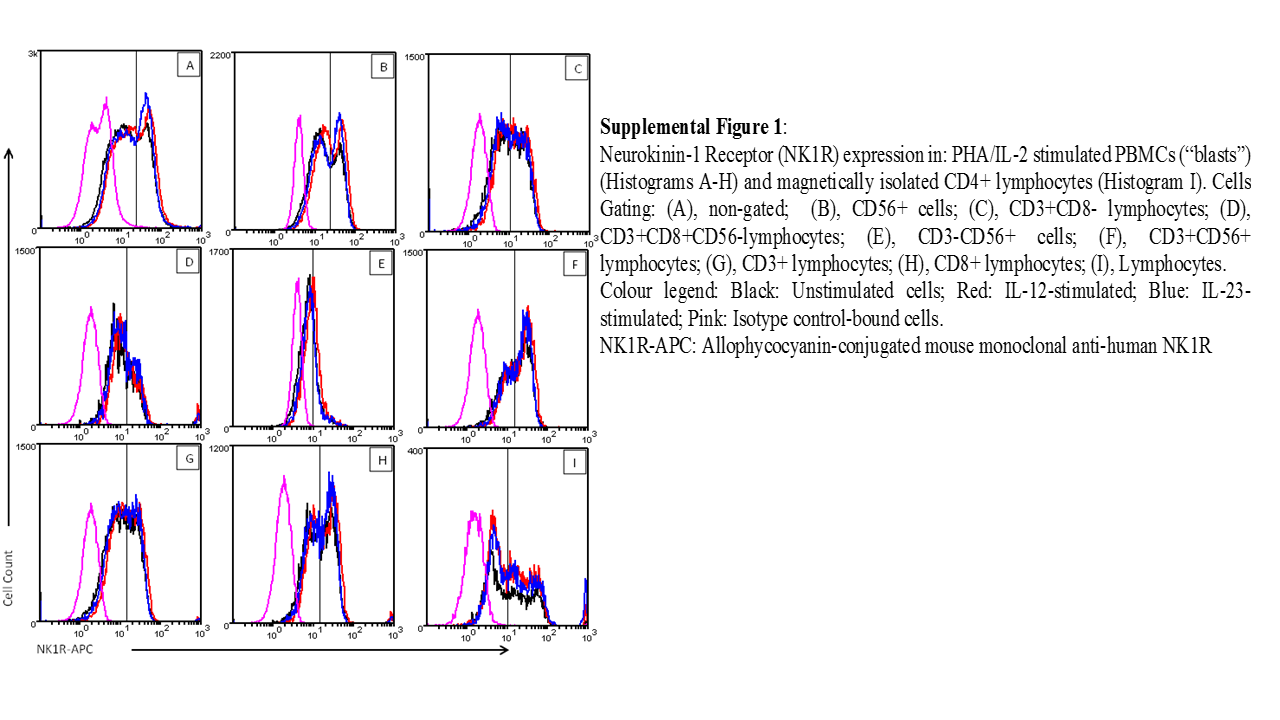

Supplement: Supplementary file 3 — (TIFF 320 kb) [file 11481_2015_9589_MOESM2_ESM.tif]

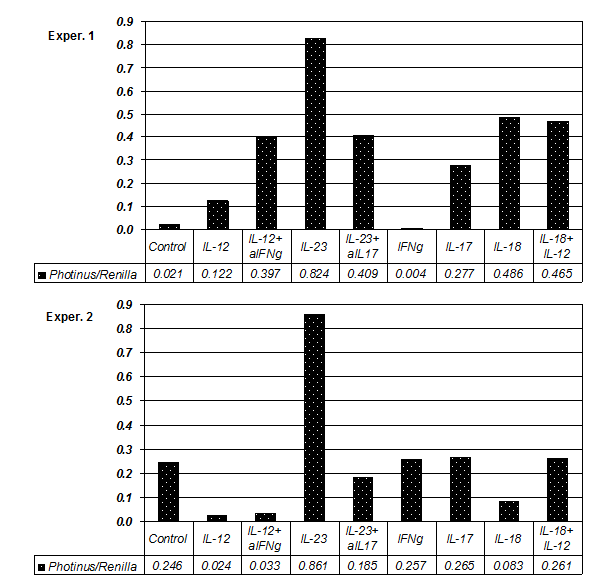

Supplement: Supplementary file 5 — (TIFF 36 kb) [file 11481_2015_9589_MOESM3_ESM.tif]
